# Smartphone, Social Media, and Mental Health App Use in an Acute Transdiagnostic Psychiatric Sample

**DOI:** 10.2196/13364

**Published:** 2019-06-07

**Authors:** Courtney Beard, Alexandra L Silverman, Marie Forgeard, M Taylor Wilmer, John Torous, Thröstur Björgvinsson

**Affiliations:** 1 McLean Hospital/Harvard Medical School Belmont, MA United States; 2 William James College Newton, MA United States; 3 Beth Israel Deaconess Medical Center/Harvard Medical School Boston, MD United States

**Keywords:** mobile health, smartphone, social media, serious mental illness

## Abstract

**Background:**

Despite high rates of smartphone ownership in psychiatric populations, there are very little data available characterizing smartphone use in individuals with mental illness. In particular, few studies have examined the interest and use of smartphones to support mental health.

**Objective:**

This study aimed to (1) characterize general smartphone app and social media usage in an acute transdiagnostic psychiatric sample with high smartphone ownership, (2) characterize current engagement and interest in the use of smartphone apps to support mental health, and (3) test demographic and clinical predictors of smartphone use.

**Methods:**

The survey was completed by all patients attending an adult partial hospital program, with no exclusion criteria. The primary outcomes were frequency of use of general and mental health smartphone apps (smartphone use survey) and the frequency of social media use and phone-checking behavior (mobile technology engagement scale).

**Results:**

Overall, 322 patients (aged mean 33.49, SD 13.87 years; 57% female) reported that their most frequently used app functions were texting, email, and social media. Younger individuals reported more frequent use across most types of apps. Baseline depression and anxiety symptoms were not associated with the frequency of app use. Participants reported health care, calendar, and texting apps as most supportive of their mental health and social media apps as most negatively affecting their mental health. Most patients reported an interest in (73.9% [238/322]) and willingness to use (81.3% [262/322]) a smartphone app to monitor their mental health condition. Less than half (44%) of the patients currently had a mental health app downloaded on their smartphone, with mindfulness and meditation apps being the most common type.

**Conclusions:**

The high interest in and willingness to use mental health apps, paired with the only moderate current reported usage, indicate a potential unmet treatment opportunity in psychiatric populations. There is potential to optimize non-mental health–specific apps to better support the needs of those with mental illness and to design a new wave of mental health apps that match the needs of these populations as well as the way they use smartphones in daily life.

## Introduction

Estimates from 2018 suggest that 77% of the US population owns a smartphone [1]. The availability of smartphone apps designed to enhance wellness or support mental health has also grown exponentially, with over 10,000 available [2]. Studies document a strong interest in using smartphones to promote mental health in individuals with mental illness. In a meta-analysis of individuals with psychosis, 60% endorsed interest in using their phone to monitor their mental health [3]. In addition, over half of them were interested in using their phones to obtain health care information, receive appointment or medication reminders, and facilitate contact with providers. Approximately 30% of participants recruited from community mental health centers reported using apps for enhancing physical wellness, primarily to support exercise, diet, and weight loss [4], although participants were not queried about using apps for specific mental health purposes. Individuals receiving outpatient psychiatric care report similar levels of interest in using smartphones to track mental health (56% to 84%) [5-7].

Beyond this handful of studies, most of the existing data on smartphone use in individuals with mental illness focus on social media. Reports of social media use among individuals with serious mental illness (SMI) range from 33% to 71%, with Facebook and Twitter identified as the most frequently used platforms [4,8-10]. Most users reported engaging with social media daily, with higher use for younger individuals [4,9-12]. Individuals with SMI reported using social media for reasons such as interacting with friends, family, or others outside the home as well as for searching for health-related information; in addition, interest in mental health programs delivered through social media was high [4,8,10,12].

Popular media has highlighted growing concern about the potential addictive nature of smartphone and social media use as well as the subsequent negative mental health effects. Indeed, problematic smartphone use has consistently been associated with higher levels of depression and anxiety in nonclinical samples [13,14], and greater time spent on social media was correlated with higher depression among individuals receiving inpatient psychiatric treatment [15]. In contrast, other studies indicate that individuals with mental illness disagree that smartphone and social media use necessarily negatively impact their lives. In 1 study, half of the individuals with schizophrenia agreed that social media use increased socialization and disagreed that it made their symptoms worse [10]. In a community clinic sample, social media use was not associated with the severity of psychiatric symptoms or quality of life, but instead, it was positively associated with greater community participation and civic engagement [9]; although in other studies, results have been more mixed [16]. Thus, although the potential clinical risks of smartphone and social media use warrant close monitoring and further study, individuals may also perceive benefit.

Overall, existing data suggest that individuals with mental illness increasingly own smartphones, regularly use social media, and express an interest in using smartphones and social media to support their health (though potential negative consequences also need to be considered). However, there remain several important gaps in this nascent field. First, most studies that have examined how individuals with mental illness use smartphone apps to promote their health have not focused on mental health specifically [4]. Second, although some initial studies investigated demographic and clinical correlates of smartphone *ownership*, only 1 study has characterized *use* in this manner, and it focused specifically on social media [9]. Finally, some previous studies pertaining to smartphone interest may be positively skewed. For example, in some studies, as few as 10% of patients at a clinic chose to complete the study surveys [5,6], and others surveyed individuals who were already volunteering for another study about a smartphone app [17]. Thus, studies assessing smartphone use are needed in larger naturalistic samples in which individuals complete measures as part of routine care and whose results therefore should be less affected by selection biases.

The main aim of this study was to provide a detailed account of general smartphone use (including social media) in individuals with mental illness. The secondary aim was to better understand current engagement with mental health apps specifically and to assess interest in using smartphone apps to support mental health. The results may inform efforts to harness digital technologies for delivering mental health interventions and guide research, clinical, and industry efforts [4,8,16,18]. To that end, we administered self-reported measures of smartphone use, social media use, and interest in mental health apps to all individuals attending a psychiatric partial hospital program. Given the demographic characteristics of this partial hospital program, we expected a very high rate of smartphone ownership; thus, making the sample well suited to explore patterns of use and interest in smartphone apps. On the basis of previous studies, we expected high rates of use across all 3 domains. We explored demographic and clinical correlates of smartphone use and expected that younger individuals would report higher use than older individuals [4,9,10,12]. The remaining analyses were descriptive and exploratory given the lack of previous research focused on these specific questions.

## Methods

### Participants and Treatment Setting

Participants included 322 patients receiving treatment at a partial hospitalization program (PHP) located in a nonprofit, insurance-based, academic psychiatric hospital in New England from September 2017 to March 2018. The PHP treats English-speaking adults (≥18 years) with a broad range of psychiatric disorders [19]. Participants were on average in young-to-middle adulthood (mean 33.49, SD 13.87 years), female (57%), and predominantly white (89%). Approximately half of the patients are referred directly from an inpatient hospitalization for further stabilization and transition to outpatient care. The other half of them are referred from outpatient providers in lieu of an inpatient hospitalization. Thus, to qualify for a partial hospital level of care, individuals are experiencing acute symptoms and significant impairment. Many patients are managing chronic mental illness, whereas others may be experiencing their first episode. The most common primary diagnosis (provided by program psychiatrists) was major depressive disorder (MDD; n=183), followed by bipolar disorder (n=72); anxiety, obsessive-compulsive disorder, or stress-related disorders (n=48); and psychotic disorder (n=19). See Table 1 for demographic characteristics.

**Table 1:** Demographic and clinical characteristics.

Demographic characteristic		Values
Age, years (mean, SD)		33.49 (13.87)
**Gender, n (%)**		
	Female	183 (56.8)
	Male	135 (41.9)
	Gender fluid or nonbinary	4 (1.2)
**Race**		
	White	287 (89.1)
	Asian	12 (3.7)
	Multiracial	11 (3.4)
	Black	6 (1.9)
	Did not specify	6 (1.9)
**Ethnicity**		
	Non-Latinx	300 (93.2)
	Latinx	21 (6.5)
	Did not specify	1 (0.3)
**Sexual** **orientation**		
	Heterosexual/straight	248 (77.0)
	Bisexual	33 (10.3)
	Gay/lesbian	18 (5.6)
	Queer	11 (3.4)
	Something else (asexual and pansexual)	12 (3.7)
**Education**		
	High school/GED or less	17 (5.3)
	Some college	120 (37.3)
	4-year college graduate	91 (28.3)
	Postcollege education	94 (29.2)
	Current student	99 (30.7)
**Employment**		
	Not employed	153 (47.5)
	Employed part time	57 (17.7)
	Employed full time	112 (34.8)
**Marital** **status**		
	Never married	206 (64.0)
	Separated/divorced or widowed	31 (9.6)
	Married	72 (22.4)
	Living with partner	13 (4.0)

### Measures

#### Smartphone Use Survey

We developed a self-reported questionnaire to assess smartphone ownership, application usage, and interest in applications to support mental health based on a previous survey assessing smartphone usage in psychiatric outpatients [5,6]. The survey also asked participants to indicate which smartphone apps they perceived as “supportive of” versus “negatively affecting” their mental health (see Multimedia Appendix 1).

#### Mobile Technology Engagement Scale

The mobile technology engagement (MTE) [20] is a self-reported measure of mobile technology usage. First, the *social media usage* component consists of 4 items assessing mobile use of social media platforms, including Facebook, Twitter, Instagram, and Snapchat, with each item anchored on a 7-point Likert-style scale. The social media usage score is calculated by summing the 4 items. Second, the *status updates* component consists of a single item (“How often do you post public updates—Facebook status, Tweets, Instagram uploads, etc”) using a 7-point Likert-style scale, with the raw score indicating a status updates score. Third, the *phone-checking behavior* component consists of 3 items assessing relevant behaviors, with response options anchored on a 5-point Likert-style scale. The phone-checking behavior score is the mean of the 3 phone-checking behavior items. To calculate overall MTE scores, a z-score is calculated for social media usage, status updates, and phone-checking behavior, and then the mean of the z-scores is used as an individual’s overall score, with higher scores indicating higher engagement. Internal consistency was acceptable (Cronbach alpha=.71).

#### Patient Health Questionnaire-9

The Patient Health Questionnaire-9 (PHQ-9) [21] is a self-reported measure that assesses the frequency of depressive symptoms over the past 2 weeks using a 4-point Likert-type scale anchored at 0 (*not at all*) and 3 *(nearly all the time*). A total PHQ-9 score is calculated by summing items, with higher scores indicating higher depression severity. The PHQ-9 has demonstrated strong psychometric properties in similar psychiatric populations [22]. In this sample, internal consistency was good (Cronbach alpha=.86).

#### Generalized Anxiety Disorder Scale—7 items

The Generalized Anxiety Disorder Scale—7 items (GAD-7) [23] is a self-reported questionnaire that assesses symptoms of generalized anxiety disorder over the past 2 weeks (0, not at all, to 3, nearly every day), with higher scores indicating greater anxiety severity. The GAD-7 has been validated as a measure of symptoms of general anxiety in a psychiatric hospital setting [23-26]. In this sample, internal consistency was good (alpha=.88).

#### Clinical Global Improvement Scale

The Clinical Global Improvement Scale [27] is a single item assessing patients’ perceived improvement (or lack thereof) compared with their baseline status. In this study, patients rated their own improvement at discharge using the 7-point scale from 1, very much improved, to 7, very much worse. Patient ratings correlate moderately with provider ratings (Intraclass Correlation Coefficient=.65) and have comparable validity [28].

### Procedure

As part of routine clinical care at the PHP, patients completed self-reported measures of symptoms and functioning. Patient data were originally used by their treatment providers for treatment planning and progress monitoring. Patient data are also used for program evaluation and quality assurance efforts. The current measures of smartphone use were included to inform ongoing treatment development and evaluation efforts at the PHP and, thus, did not require informed consent. Measures were administered using REDCap (Research Electronic Data Capture), a secure, Web-based application designed to support data collection for research studies [29].

We obtained a deidentified dataset from the PHP, and the local institutional review board deemed this research exempt. In this dataset, 10.9% of participants did not complete the discharge assessment for the following reasons: they were admitted to inpatient hospitalization (n=17), they unexpectedly did not attend the program on the day of discharge (n=14), or staff were unable to schedule assessment (n=4).

#### App Standardization and Categorization

We followed similar procedures as previously described [17]. We standardized the names of each mental health app. For example, Headspace, head space, and HeadSpace were all coded as the same app. We then categorized the most frequently reported apps according to their advertised purpose. To do this, 1 author (ALS) first generated potential categories based on the description in the Google Play store. Second, ALS and CB discussed the potential categories and came to a consensus about the final list as well as how each app was categorized (see Multimedia Appendix 2).

#### Data Analytic Strategy

We calculated descriptive statistics (frequency, %) for the smartphone use items. To facilitate comparisons with previous studies, we present the responses stratified by the age groups used by Torous et al (see Table 2) [5]. We explored whether demographic characteristics (age, gender, education, and race) and clinical characteristics (baseline depression and anxiety symptom severity, primary diagnosis [bipolar, MDD, anxiety, and psychotic], and treatment response [reported 1, very much, or 2, much improvement, vs other responses]) were associated with responses on the smartphone use survey and MTE. To simplify interpretation of results, we transformed the education variable from its 5 response options to a dichotomous variable (4-year college degree or more vs no college degree). Owing to the small number of individual racial categories endorsed, we used participants’ responses (yes/no) to the *white* racial category to examine race.

**Table 2:** Frequency of smartphone app use in total sample and by age group.

Type of app		Never, n (%)	Rarely, n (%)	Sometimes, n (%)	Frequently, n (%)	Often, n (%)	Very often, n (%)
**Texting apps (eg, WhatsApp)**							
	Total sample	18 (5.7)	6 (1.9)	13 (4.1)	33(10.5)	80 (25.4)	165 (52.4)
	Under 30 years	5 (2.9)	3 (1.7)	7 (4)	14 (8.1)	39 (22.5)	105 (60.7)
	31-45 years	6 (8)	2 (2)	3 (4)	8 (10)	24 (31)	34 (44)
	46-60 years	5 (10)	0 (0)	3 (6)	7 (14)	12 (24)	24 (47)
	Over 60 years	2 (14)	1 (7)	0 (0)	4 (29)	5 (36)	2 (14)
**Phone/video apps (eg, Skype)**							
	Total sample	48 (15.3)	84 (26.8)	58 (18.5)	49 (15.7)	43 (13.7)	31 (9.9)
	Under 30 years	18 (10.3)	41 (23.6)	34 (19.5)	31 (17.8)	29 (16.7)	21 (12.1)
	31-45 years	15 (20)	21 (28)	14 (18)	11 (15)	9 (12)	6 (8)
	46-60 years	12 (25)	16 (33)	8 (16)	7 (14)	3 (6)	3 (6)
	Over 60 years	3 (21)	6 (43)	2 (14)	0 (0)	2 (14)	1 (7)
**Email apps (eg, Outlook)**							
	Total sample	3 (.9)	9 (2.9)	22 (7)	45 (14.3)	111 (35.2)	125 (39.7)
	Under 30 years	0 (0)	7 (4)	16 (9.2)	34 (19.5)	58 (33.3)	59 (33.9)
	31-45 years	1 (1)	1 (1)	3 (4)	7 (9)	30 (39)	35 (46)
	46-60 years	2 (4)	1 (2)	1 (2)	4 (8)	15 (30)	27 (54)
	Over 60 years	0 (0)	0 (0)	2 (14)	0 (0)	8 (57)	4 (29)
**Social media apps (eg, Facebook**)							
	Total sample	43 (13.8)	17 (5.4)	31 (9.9)	32 (10.3)	66 (21.2)	123 (39.4)
	Under 30 years	11 (6.4)	4 (2.3)	20 (11.6)	16 (9.2)	40 (23.1)	82 (47.4)
	31-45 years	17 (23)	4 (5)	3 (4)	9 (12)	17 (23)	25 (33)
	46-60 years	11 (22)	6 (12)	5 (10)	4 (8)	8 (16)	16 (32)
	Over 60 years	4 (29)	3 (21)	3 (21)	3 (21)	1 (7)	0 (0)
**Calendar apps**							
	Total sample	46 (14.8)	30 (9.7)	41 (13.2)	54 (17.4)	83 (26.8)	56 (18.1)
	Under 30 years	23 (13.4)	23 (13.4)	31 (18)	35 (20.3)	38 (22.1)	22 (12.8)
	31-45 years	11 (15)	5 (7)	6 (8)	12 (16)	22 (29)	20 (26)
	46-60 years	10 (21)	2 (42)	3 (6)	4 (8)	18 (38)	11 (23)
	Over 60 years	2 (14)	0 (0)	1 (7)	3 (21)	5 (36)	3 (21)
**Entertainment apps (eg, YouTube and radio)**							
	Total sample	28 (8.9)	41 (13)	56 (17.7)	62 (19.6)	72 (22.8)	57 (18)
	Under 30 years	14 (8)	17 (9.8)	22 (12.6)	35 (20.1)	45 (25.9)	41 (23.6)
	31-45 years	7 (9)	6 (8)	16 (21)	16 (21)	19 (25)	13 (17)
	46-60 years	6 (12)	14 (28)	12 (24)	8 (16)	8 (16)	3 (6)
	Over 60 years	1 (7)	4 (29)	6 (43)	3 (21)	0 (0)	0 (0)
**Games**							
	Total sample	123 (39.7)	57 (18.4)	37 (11.9)	39 (12.6)	28 (9)	26 (8.4)
	Under 30 years	60 (34.7)	32 (18.5)	21 (12.1)	24 (13.9)	19 (11.0)	17 (9.8)
	31-45 years	31 (40)	17 (22)	13 (17)	7 (9)	6 (8)	3 (4)
	46-60 years	23 (49)	7 (15)	2 (4)	7 (15)	2 (4)	6 (13)
	Over 60 years	9 (69)	1 (8)	1 (8)	1 (8)	1 (8)	0 (0)

We conducted multiple ordinal logistic regression analyses with the 6 levels of frequency of use as the dependent variable (never, rarely, sometimes, frequently, often, and very often) and demographic and clinical variables as the predictors. All the odds ratios presented represent the effect of the predictor on the dependent variable holding all other predictor variables in the model constant. For each of these models, we tested the assumption of proportional odds (that the coefficients are equal across all levels) using the test of parallel lines in SPSS, and we report below whether this assumption was violated in any models.

We conducted multiple logistic regressions with interest and willingness to use apps to monitor mental health as the dependent variables (yes/no) and demographic and clinical variables as the predictors. Finally, we conducted multiple linear regression analyses to examine the demographic and clinical predictors of the number of mental health apps on patients’ phones and MTE subscales.

## Results

### Aim 1: General Smartphone App and Social Media Use

Almost all the patients reported owning a smartphone (n=315/322, 98%): iPhone (n=250/322, 77.7%) and Android (n=65/322, 20.2%). The most frequently used types of apps were texting, email, and social media (see Table 2). Multimedia Appendix 3 presents the results for the MTE scale. Patients most commonly reported using Facebook, followed by Instagram, Snapchat, and Twitter.

### Demographic and Clinical Associations

Overall, 3 of the models predicting frequency of the app use violated the assumption of proportional odds: calendar apps (*χ*^2^_40_ 68.8; *P*=.003), games (*χ*^2^_40_=91.4; *P*<.001), and health and mental health apps (*χ*^2^_40_=56.9; *P*=.04). Thus, use caution when interpreting the effects for predicting use of those specific apps. As expected, older age was associated with less frequent use of most apps: texting (OR 0.97, 95% CI 0.95-0.98; *P*<.001), phone/video (OR 0.97, 95% CI 0.95-0.98; *P*<.001), social media (OR 0.95, 95% CI 0.93-0.97; *P*<.001), entertainment (OR 0.96, 95% CI 0.94-0.98; *P*<.001), and games (OR 0.98, 95% CI 0.96-1.00; *P*=.04).

Gender also predicted the use of several types of apps, with women reporting more frequent use of social media (OR 1.92, 95% CI 1.21-3.05; *P*=.006) and physical and mental health apps (OR 1.67, 95% CI 1.05-2.64; *P*=.03) but less frequent use of entertainment apps (OR 0.49, 95% CI 0.31-0.76; *P*=.002) compared with men. Lower education was associated with less frequent use of email (OR 0.36, 95% CI 0.21-0.60; *P*<.001), calendar (OR 0.46, 95% CI 0.29-0.76; *P*=.002), and entertainment (OR 0.59, 95% CI 0.36-0.95; *P*=.03) apps. Finally, participants of color reported less frequent use of social media (OR 0.28, 95% CI 0.12-0.65; *P*=.003).

Regarding the clinical characteristics, absence of a primary diagnosis of MDD was associated with less frequent use of phone/video communication (OR 0.29, 95% CI 0.10-0.82; *P*=.02). Primary diagnosis was also associated with the use of entertainment apps, such that individuals who were not diagnosed with MDD (OR 0.29, 95% CI 0.10-0.80; *P*=.02), bipolar disorders (OR 0.29, 95% CI 0.10-0.83; *P*=.02), or anxiety disorders (OR 0.21, 95% CI 0.06-0.66; *P*=.008) used entertainment apps less frequently than individuals with these diagnoses. None of the other clinical variables (symptom severity and treatment responder) were associated with the frequency of app use.

Table 3 presents the regression models predicting the MTE subscales from demographic and clinical characteristics. More frequent social media use was predicted by younger age, female gender, white race, and not having a primary diagnosis of psychotic disorder (all *P*<.05). More frequent phone-checking behavior was predicted by younger age and greater anxiety severity. The only significant predictor of more frequent status updates was a primary diagnosis of bipolar disorder.

**Table 3:** Multiple regression analyses predicting mobile technology engagement subscales.

Variable	Social media usage^a^			Phone-checking^b^ behavior			Status updates^c^		
	*B* ^d^	Standard Error *B*	Beta	*B*	Standard error *B*	Beta	*B*	Standard Error *B*	Beta
Age (years)	−.032	0.004	−.444^e^	−.026	0.004	−.359^e^	−.005	0.005	−.070
Sex (male)	−.233	0.108	−.115^f^	0.127	0.114	0.063	−.179	0.12	−.091
Race (white)	0.422	0.203	.110^f^	0.394	0.215	0.103	−.077	0.225	−.021
Education	−.099	0.118	−.049	0.025	0.125	0.012	−.169	0.131	−.086
Baseline anxiety	0	0.013	−.002	0.028	0.014	.154^f^	0.008	0.015	0.045
Baseline depression	0.021	0.013	0.118	0.018	0.013	0.105	0.002	0.014	0.012
Primary bipolar disorder	−.032	0.134	−.013	−.026	0.142	−.011	0.412	0.149	.175^e^
Primary anxiety	−.015	0.17	−.005	−.111	0.18	−.036	0.045	0.189	0.015
Primary psychosis	−.676	0.25	−.150^g^	−.315	0.264	−.070	−.264	0.277	−.060
Treatment responder (yes)	0.21	0.115	0.097	0.103	0.121	0.048	0.2	0.127	0.095

^a^R^2^=.293

^b^R^2^=.204

^c^R^2^=.075

^d^*B*: Unstandardized beta.

^e^*P*<.001.

^f^*P*<.05.

^g^*P*<.01.

### Aim 2: Interest in and Use of Apps for Mental Health

Most participants reported they would want (n=238, 74%) and be willing (n=262, 81%) to use an app to monitor their mental health. Education was the only predictor of both interest (beta=−.69, SE=.31; *P*=.02; OR 0.50, 95% CI 0.28-0.91) and willingness (beta=−.73, SE .34; *P*=.03, OR 0.48, 95% CI 0.25-0.94) to use a smartphone app to monitor a mental health condition. The more educated group had higher rates of interest and willingness (79% and 85%, respectively) than the less educated group (67% and 77%, respectively).

**Figure 1:**
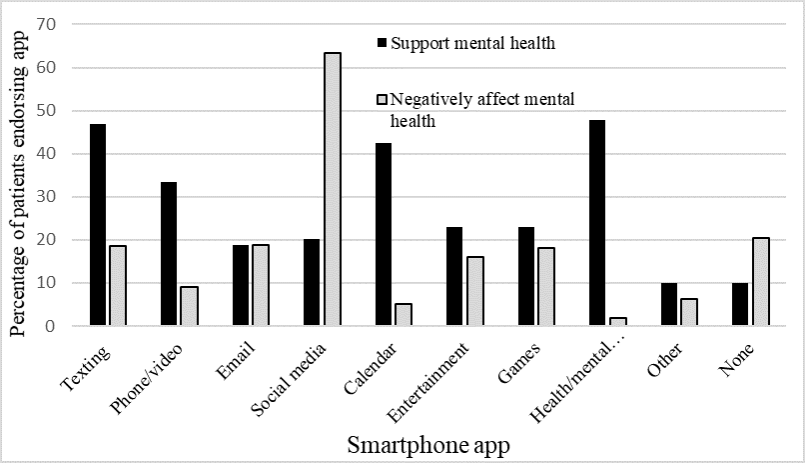
Perceived helpfulness of smartphone apps for mental health.

Perceived helpfulness of general smartphone apps for mental health. Figure 1 presents the percentage of participants who endorsed the helpfulness and unhelpfulness of each app. The apps most frequently endorsed as being supportive of mental health were texting (47%) and calendars (43%). Few participants endorsed any apps as being harmful to their mental health, except for social media (63%).

### Current Mental Health App Use

Approximately half of the patients (139/315, 44%) reported that they currently had at least 1 mental health app installed on their phone. None of the demographic or clinical characteristics significantly predicted the number of mental health apps downloaded (*P*>.08). Participants selected the primary purpose of their mental health apps as meditation/mindfulness (71%), mood tracking (10%), therapy skills (4%), and other (15%). The most commonly reported apps were Headspace (n=46), Calm (n=21), and Insight Timer (n=11; see Multimedia Appendix 2). These 3 apps were all coded as facilitating the practice of mindfulness/meditation. The 50+ other apps reported were used by 1 to 2 participants. Approximately half of the participants (154/322, 48%) endorsed health and mental health apps as being supportive of their mental health.

## Discussion

### Aim 1: General Smartphone App and Social Media Use

The most frequently used types of apps were texting, email, and social media. As expected and consistent with previous work, younger individuals reported more frequent smartphone use across most types of apps [4,9,10,12]. Education also predicted the frequency of use for some types of apps, specifically email, calendar, and entertainment apps. Baseline depression and anxiety severity were not associated with smartphone use nor was treatment response.

Regarding social media–specific behaviors, Facebook was the most commonly used social media app, followed by Instagram, Snapchat, and Twitter. This pattern aligns closely with previous research in similar samples [4,9,11,12]. Most patients reported checking their phone at least once per hour but posting rarely, potentially suggesting that our sample may primarily use social media passively and perhaps experience it as a platform for social comparison rather than for social connection or support [30,31]. Men and people of color reported less frequent use of social media. Younger individuals and those with greater anxiety checked their phones with greater frequency, and individuals with bipolar disorder made more frequent status posts relative to individuals with other primary diagnoses.

### Aim 2: Interest in and Use of Apps for Mental Health

Most patients reported an interest (74%) and willingness (81%) to use a smartphone app to monitor their mental health. Overall, the rates of interest and willingness are slightly higher than those reported by individuals attending an outpatient psychiatric clinic [5]. In that study, 67% of patients expressed an interest in using a mobile app to track their medical condition (not mental health specifically) daily [5]. Although interest and willingness were high across education level, individuals with a higher level of education reported greater interest and willingness. This finding has implications for studies developing smartphone-delivered interventions; researchers should attend to educational background when developing and advertising such interventions. Specifically, although technology-driven interventions have the potential to reduce barriers to mental health treatment, they might also unintentionally further existing disparities in access. Future research should examine whether specific efforts are needed to encourage/assist individuals with lower levels of education to use smartphone app interventions for mental health purposes.

In contrast to the high rates of reported interest in mental health apps, only 44% of patients reported currently having a mental health app downloaded on their smartphone. This rate is higher than that obtained in a recent study of individuals with anxiety and depression enrolled in a smartphone treatment study (26%) [17] and that in another study of psychiatric outpatients (10%) [32]. Participants who currently had mental health apps downloaded on their phones reported that they primarily used them for mindfulness or meditation (71%), with mood tracking being a distant second (10%). The uses of the self-reported app mirror the coded purposes of the most frequently reported mental health apps. Specifically, the top 3 apps reported (Headspace, Calm, and Insight Timer) are all designed to facilitate the practice of mindfulness/meditation.

Participants endorsed using general smartphone apps to support their mental health. Specifically, participants rated health care, calendar, and texting apps as the most supportive of their mental health, whereas most participants rated social media apps as negatively affecting their mental health. However, the frequency of social media use was not associated with symptom severity or treatment response in this study. Nevertheless, patients’ subjective report that social media harms their mental health contrasts with results of previous research, in which individuals with SMI endorsed using social media for activities related to positive mental health, such as sharing things about themselves and feeling less lonely [9]. A recent systematic review suggested that different patterns of social media engagement may have varying impacts on mental health [33]. Experiences of social connectedness and support through social media were associated with positive mental health outcomes, whereas experiences of social comparison and rumination were associated with negative mental health outcomes. It may be that individuals in our sample are more prone to ruminative response styles and more likely to engage in social comparisons through social media, thereby perceiving a negative impact of social media on their mental health [30-31]. Individuals and societal perceptions of social media may also be quickly evolving as individuals are no longer discovering these apps but instead trying to figure out whether/how to use them in the long run. Future research in this area should carefully consider both the positive and negative clinical impacts of smartphone interventions, given the potential for addiction and negative influence on internalizing symptoms.

Together, the high interest in and perceived benefit of mental health apps, but only moderate current use, suggests a potential unmet treatment opportunity. These findings support ongoing efforts to develop and validate apps that support mental health. The naturalistic use of calendar and texting apps suggests that mental health care apps that leverage these types of functions might be especially feasible and acceptable in this population. These findings also support the development of methods for helping individuals identify existing evidence-based mental health care apps that might benefit them. Like matching an appropriate therapy or medication to the right individual is a personalized decision, there are ongoing efforts to support informed decision making around mental health apps and to help both clinicians and consumers identify safe, effective, engaging, and interoperable apps [34].

Although it is not a focus of this study, some of our findings provide support for ongoing efforts toward utilizing phone use behaviors (eg, number of social media posts, number of phone calls made, and Global Positioning System location,) in detecting mental illness phenotypes, deterioration, or relapse. Specifically, we found that a diagnosis of bipolar disorder predicted more frequent social media status updates and anxiety severity predicted more frequent phone-checking behavior. These effects mirror recent studies designed to identify personalized, objective markers of relapse via passively obtained indicators of smartphone behavior [35-36]. It might be helpful for clinicians and patients to attend to these objective indicators as part of relapse management. Individual monitoring of some phone use behaviors has become easy. For example, Apple iPhone users can monitor their phone use (eg, number of messages) through the Screen Time feature in Settings, and Android users can use apps such as Quality Time for the same purpose. Such monitoring may ultimately provide significant clinical benefit for some patients, especially if confidential methods for sharing data and alerting clinicians are developed.

### Strengths and Limitations

All patients attending the psychiatric hospital program completed the measures. Thus, our results will likely generalize to other acute psychiatric populations and not only to individuals who choose to complete a survey on smartphone use. However, the results must be interpreted in the context of the hospital’s demographic makeup, which is generally well educated and lacking ethnoracial diversity. In addition, although the high-smartphone ownership rate indicates that this sample was particularly well suited to examine smartphone use (ie, it would be impossible for people who do not own smartphones to answer most of the questions we asked), it also suggests that our findings may not generalize to other settings in which ownership is less common. Thus, these results regarding the frequency of use and interest in mobile health apps should be interpreted in the context of our sample of individuals who own smartphones rather than from the general population of individuals with mental illness. In addition, because of the need to minimize participant burden, we could not assess all potentially relevant variables. For example, it would have been helpful to assess the types of data plans and how these affected app use. Finally, we were unable to independently verify smartphone use, and future studies should include objective assessments.

### Conclusions

People with mental illness increasingly have access to smartphones and are interested in apps to support mental health. However, the lower uptake of mental health apps today suggests the challenge of translating ongoing research on digital mental health into real-world tools that patients can easily access. This study suggests that among smartphone owners, there is high interest in using smartphone apps to improve mental health. Many nonmental health–specific apps (eg, calendars and texting) are perceived as beneficial for mental health, whereas social media apps are perceived as harmful to mental health. There is potential to optimize nonmental health–specific apps to better support the needs of those with mental illness and to design a new wave of mental health apps that match the needs of this population and the way they use these devices in daily life.

## Appendix

Multimedia Appendix 1 Smartphone use survey.

Multimedia Appendix 2 App names and categories.

Multimedia Appendix 3 Responses to mobile technology engagement.

## Abbreviations

GAD: Generalized Anxiety Disorder Scale
MDD: major depressive disorder
MTE: mobile technology engagement
PHP: partial hospitalization program
PHQ: Patient Health Questionnaire
SMI: serious mental illness
