# Supplementary material for: Smartphone, Social Media, and Mental Health App Use in an Acute Transdiagnostic Psychiatric Sample
Source: JMIR Mhealth Uhealth. 2019 Jun 7;7(6):e13364. doi: 10.2196/13364 (PMC6592519; doi:10.2196/13364)
Supplement: Multimedia Appendix 1 [file mhealth_v7i6e13364_app1.docx]

Appendix A

Smartphone Use Survey

1. What type of phone do you use?

I don’t use a mobile phone [SKIP TO ITEM 6]

Flip phone or older phone (i.e., not a smart phone) [SKIP TO ITEM 6]

Apple iPhone

Android (e.g., Samsung, LG, HTC, Google phone, etc.)

Other (specify)

2. How often do you use the following types of applications or “apps”?

Never, Rarely (Once a month), Sometimes (Once a week), Frequently (Several times a week), Often (Daily), Very often (Several times a day)

2a. Texting apps (e.g., Messenger, Whatsapp)

2b. Phone/Video communication apps (e.g., Skype, Facetime, Hangouts, Facebook Messenger)

2c. Email apps (e.g., Outlook, Gmail, etc.)

2d. Social media apps (e.g., Facebook, Twitter, Snapchat, Instagram, etc.)

2e. Calendar apps

2f. Entertainment apps (e.g., Podcasts, Stitcher, Youtube, Radio, etc.)

2g. Games

2h. Health and mental health apps

2i. Other apps

3. Which of these apps do you think SUPPORT your mental health (i.e., you notice using them in a way that benefits your mental health)? Check all of that apply.

Checkbox options: [None, Texting apps, Phone/video communication apps, Email apps, social media apps, calendar apps, entertainment apps, games, health and mental health apps, other apps]

4. Which of these apps do you think NEGATIVELY AFFECT your mental health (i.e., you notice that using them results in lowering your well-being). Check all that apply.

Checkbox options: [None, Texting apps, Phone/video communication apps, Email apps, social media apps, calendar apps, entertainment apps, games, health and mental health apps, other apps]

5. How many mental health care related applications or “apps” do you have on your phone?

Dropdown menu: 0, 1, 2, 3, 4, 5, 6, 7, 8, 9, 10, 10+

5a. What mental health apps do you use regularly? Please list the name(s) of the app(s)

5b. What do you most often use mental health apps for?

Mood tracking

Safety planning

Meditation/mindfulness/ Breathing exercises

Therapy Skills

Communicating with a therapist

Other

6. Would you want to use a Smartphone app to help track your mental health condition?

Yes No

7. Would you be willing to use a Smartphone app on a daily basis to help monitor your mental health condition?

Yes No
