# Supplementary material for: Smartphone, Social Media, and Mental Health App Use in an Acute Transdiagnostic Psychiatric Sample
Source: JMIR Mhealth Uhealth. 2019 Jun 7;7(6):e13364. doi: 10.2196/13364 (PMC6592519; doi:10.2196/13364)
Supplement: Multimedia Appendix 2 [file mhealth_v7i6e13364_app2.docx]

Appendix B

App Names and Categories

| **App name** | **N** | **Category** |
| --- | --- | --- |
| Headspace | 46 | Meditation/mindfulness |
| Calm | 21 | Meditation/mindfulness |
| Insight Timer | 11 | Meditation/mindfulness |
| Sleepcycle | 5 | Self-monitoring/mood tracking |
| Apple Health Center | 3 | Self-monitoring/mood tracking |
| CBT-I Coach | 3 | Therapy Skills |
| Rx reminder/walgreens | 3 | Other |
| AA | 2 | Self-monitoring/mood tracking |
| Bliss | 2 | Therapy skills |
| Breathe | 2 | Meditation/mindfulness |
| Buddhify | 2 | Meditation/mindfulness |
| DBSA | 2 | Self-monitoring/mood tracking |
| Mango Health | 2 | Other |
| Moodpath | 2 | Self-monitoring/mood tracking |
| Pacifica | 2 | Self-monitoring/mood tracking |
| Pillow | 2 | Self-monitoring/mood tracking |
| T2 Mood tracker | 2 | Self-monitoring/mood tracking |
| Weight watchers | 2 | Self-monitoring/mood tracking |
| 10% happier | 1 | Meditation/mindfulness |
| 2 Bad Habits | 1 | Other |
| 4 pics 1 word | 1 | Other |
| ACT coach | 1 | Therapy skills |
| Boosterbuddy | 1 | Therapy skills |
| CalmHarm | 1 | Therapy skills |
| Centered | 1 | Self-monitoring/mood tracking |
| Daily Quote | 1 | Other |
| Daylio | 1 | Self-monitoring/mood tracking |
| Dbt diary card | 1 | Therapy skills |
| Eating Healthy | 1 | Self-monitoring/mood tracking |
| e-moods | 1 | Self-monitoring/mood tracking |
| Fabulous | 1 | Therapy skills |
| Fitbit | 1 | Self-monitoring/mood tracking |
| Focus Keeper | 1 | Self-monitoring/mood tracking |
| Futurenda | 1 | Self-monitoring/mood tracking |
| Good Morning | 1 | Other |
| Growth Journal | 1 | Self-monitoring/mood tracking |
| Level Up | 1 | Other |
| Lifetime fitness | 1 | Other |
| Lumosity | 1 | Other |
| Mindbody | 1 | Other |
| Moment | 1 | Self-monitoring/mood tracking |
| Moodnotes | 1 | Therapy skills |
| Peak | 1 | Other |
| Pink Cloud | 1 | Other |
| Pocket Casts | 1 | Other |
| Recovery Record | 1 | Self-monitoring/mood tracking |
| Round | 1 | Other |
| RU4S | 1 | Other |
| Running for weight loss | 1 | Other |
| SAM | 1 | Therapy skills |
| Sleep Genius | 1 | Other |
| Therachat | 1 | Therapy skills |
| Thought Diary | 1 | Therapy skills |
| Whats Up? | 1 | Therapy skills |
| Yoga Nidra | 1 | Meditation/mindfulness |
| Yoga quotes | 1 | Other |
| Yoga studio | 1 | Meditation/mindfulness |
| Yoga quotes | 1 | Other |
| Yoga studio | 1 | Meditation/mindfulness |

*Note.* The ‘Other’ category includes apps designed for health and fitness, games, entertainment, meeting locators, or unknown purpose.
