# Supplementary material for: Smartphone, Social Media, and Mental Health App Use in an Acute Transdiagnostic Psychiatric Sample
Source: JMIR Mhealth Uhealth. 2019 Jun 7;7(6):e13364. doi: 10.2196/13364 (PMC6592519; doi:10.2196/13364)
Supplement: Multimedia Appendix 3 [file mhealth_v7i6e13364_app3.docx]

Multimedia Appendix 3. Responses to mobile technology engagement.

| Statistics N (%) | | | | | | | |
| --- | --- | --- | --- | --- | --- | --- | --- |
| MTE item |  |  |  |  |  |  |  |
|  | Not installed | Rarely | 5-10 min | 10-20 min | 20-40 min | 40-60 min | > 1 hour |
| Social media |  |  |  |  |  |  |  |
| Facebook | 82  (28.3) | 54  (18.6) | 45 (15.5) | 29 (10.0) | 31 (10.7) | 20  (6.9) | 29  (10.0) |
| Twitter | 189 (65.4) | 53  (18.3) | 13 (4.5) | 9  (3.1) | 7  (2.4) | 8  (2.8) | 10  (3.5) |
| Instagram | 114 (39.3) | 51  (17.6) | 27 (9.3) | 30 (10.3) | 29 (10.0) | 20  (6.9) | 19  (6.6) |
| Snapchat | 151 (52.2) | 52  (18.0) | 28 (9.7) | 18  (6.2) | 11  (3.8) | 16  (5.5) | 13  (4.5) |
|  |  |  |  |  |  |  |  |
|  | A few times | Every 2-3 hours | Every hour | 20-40 min | 10-20 min | 5-10 min | < 5 min |
| Check phone for new activity | 24  (8.3) | 23  (7.9) | 73 (25.2) | 67 (23.1) | 52 (17.9) | 41 (14.1) | 10  (3.4) |
|  |  |  |  |  |  |  |  |
|  | Rarely | Few times/month | Few times/week | 1-3/day | 3-5/day | 5-10/day | > 10/day |
| Post public updates | 175 (60.3) | 60 (20.7) | 37 (12.8) | 11  (3.8) | 3  (1.0) | 1  (.30) | 3  (1.0) |
|  |  |  |  |  |  |  |  |
|  |  | Never | Rarely | Some-times | Most of the time- wish I did it less | Most of the time- it's not a big deal |  |
| Check phone when have a few moments to spare | | 8  (2.7) | 23 (7.9) | 54 (18.6) | 111 (38.1) | 95 (32.6) |  |
| Check phone during conversations or when hanging around with friends | | 32  (11.0) | 100 (34.4) | 121 (41.6) | 32 (11.0) | 6  (2.1) |  |
